# From competition to cure: the development of live biotherapeutic products for anticancer therapy in the iGEM competition

**DOI:** 10.3389/fbioe.2024.1447176

**Published:** 2024-09-16

**Authors:** Luka Van den Berghe, Joleen Masschelein, Vitor B. Pinheiro

**Affiliations:** ^1^ Laboratory for Biomolecular Discovery and Engineering, Department of Biology, KU Leuven, Leuven, Belgium; ^2^ VIB-KU Leuven Center for Microbiology, Leuven, Belgium; ^3^ Department of Pharmaceutical and Pharmacological Sciences, Rega Institute, KU Leuven, Leuven, Belgium

**Keywords:** live biotherapeutic product, iGEM, cancer, synthetic biology, therapy

## Abstract

Cancer is a leading cause of mortality globally, often diagnosed at advanced stages with metastases already present, complicating treatment efficacy. Traditional treatments like chemotherapy and radiotherapy face challenges such as lack of specificity and drug resistance. The hallmarks of cancer, as defined by Hanahan and Weinberg, describe tumors as complex entities capable of evolving traits that promote malignancy, including sustained proliferation, resistance to cell death, and metastasis. Emerging research highlights the significant role of the microbiome in cancer development and treatment, influencing tumor progression and immune responses. This review explores the potential of live biotherapeutic products (LBPs) for cancer diagnosis and therapy, focusing on projects from the International Genetically Engineered Machines (iGEM) competition that aim to innovate LBPs for cancer treatment. Analyzing 77 projects from 2022, we highlight the progress and ongoing challenges within this research field.

## 1 Introduction

Cancer is a major cause of death worldwide and is often only diagnosed at advanced stages when secondary tumors or metastases are already present. Success of traditional cancer treatments, such as chemotherapy and radiotherapy, can be hampered by lack of specificity for tumor cells, or the emergence of drug resistance (Baban et al., 2010). The conceptual framework for understanding diverse neoplastic diseases has been established from the hallmarks of cancer proposed by Hanahan and Weinberg (2011). These hallmarks depict tumors as complex tissues consisting of diverse cell types that have the ability to evolve and acquire traits that promote proliferation and malignancy. These traits encompass sustained proliferation, evasion of growth suppressors, resistance to cell death, replicative immortality, induction of angiogenesis, and activation of invasion and metastasis. Additional enabling characteristics, such as genome instability, mutations and tumor-promoting inflammation, contribute to multistep tumor progression within the tumor microenvironment (TME). Finally, hallmarks that emerge in neoplastic tissues involve the reprogramming of energy metabolism to meet proliferative cell demands and the evasion of immune surveillance.

Recognized as a potential critical factor in cancer, the impact of the microbiome on cancer development and treatment outcomes is well-established. Villemin et al. (2023) recently reviewed the link between the microbiome and cancer, outlining potential mechanisms. Distinct microbiomes within the TME have been observed in different cancer types, influencing processes such as cancer development through the induction of interleukin (IL) 17 production (Jin et al., 2019). While certain microbiome members may induce cancer, dysbiosis has also been linked with changes in cancer progression (Lam et al., 2021). Given the substantial influence of the microbiome on cancer, there is an increasing interest in utilizing bacterial strains as diagnostic or prognostic biomarkers and exploring therapeutic approaches aimed at modifying microbiome composition. The promise of such an approach was already proven in the 19^th^ century by William Coley, who used live or heat-killed *Streptococcus pyogenes* and *Serratia marcescens* to stimulate anticancer immune responses in patients with inoperable cancers (McCarthy, 2006). More recently, these endeavors include fecal microbiota transplantation (FMT). Furthermore, our increasing understanding of the human microbiome, along with advances in synthetic biology and bioengineering, have sparked renewed interest in developing live biotherapeutic products (LBPs) for detection and treatment of cancer. Such products range from entire microbial communities to individual engineered or non-engineered bacterial strains. These bacteria can be either pathogenic or commensal (Sorbara and Pamer, 2022; Brevi and Zarrinpar, 2023) and typically include species from facultative and obligate anaerobic genera, such as *Clostridium, Bifidobacterium, Listeria, Salmonella, Escherichia, Lactococcus* and *Lactobacillus* (Kang et al., 2022).

**FIGURE 1:**
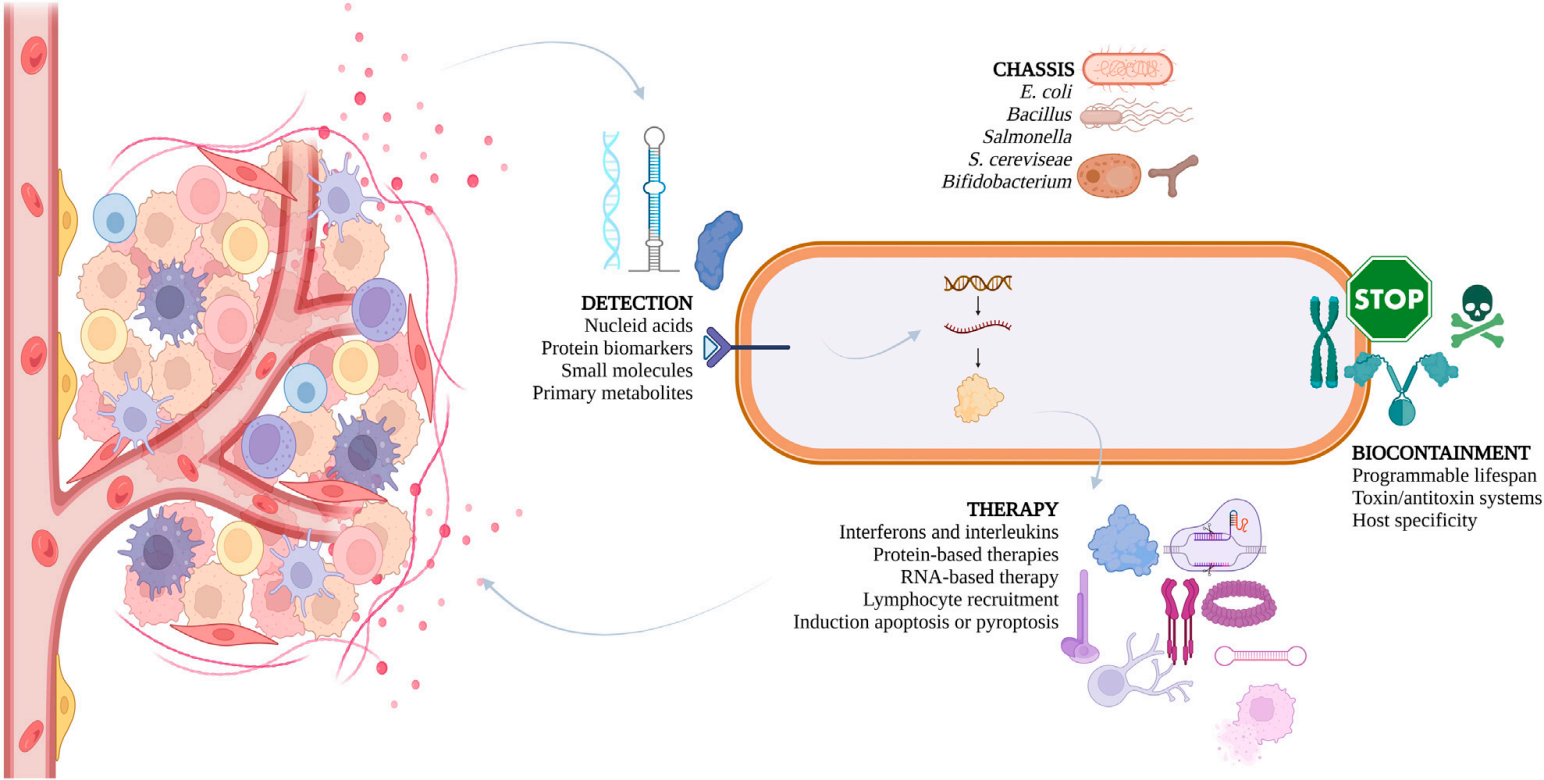
Overview of strategies used by iGEM teams over the years to develop live biotherapeutics, aiming to treat various cancers.

Nonetheless, the potential of LBPs is not restricted to the gut. After colonizing a tumor, LBPs can proliferate within the TME and trigger an anti-tumor immune response characterized by increased immune surveillance and reduced immunosuppression. However, the use of bacteria alone is often not sufficient for completely eradicating the tumor. Therefore, genetically modifying bacteria to carry therapeutic payloads has emerged as a promising new approach for cancer treatment (Nguyen et al., 2023). These engineered bacteria can be programmed to produce various therapeutic agents, such as compounds that kill tumor cells, modulate the immune system, release cytokines, activate prodrugs, interfere with RNA function, and even produce nanobodies (Duong et al., 2019). By recruiting and activating immune cells, and inducing cytokine and chemokine expression, the therapeutic payloads can play a crucial role in reshaping the TME (Duong et al., 2019) – see Figure 1.

This review centers on projects from the International Genetically Engineered Machines (iGEM) competition, specifically those dedicated to advancing live biotherapeutic products for diagnosing or treating various forms of cancer.

Using the 2022 data available on the iGEM website as well as the community-based Phoenix initiative, we selected 77 projects (of a total of 3,339 available since 2004) that harness bacteria or cell compartments for developing diagnostic and therapeutic tools against cancers (Hussain et al., 2023). Regrettably, data from 2021 projects were not available at the time of writing. We highlight here the models and the progress achieved by those teams and explore the research gaps that remain within the competition landscape. However, not all iGEM projects achieve proof-of-principle demonstration and some promising ideas could not be demonstrated within the format of the competition.

## 2 International genetically engineered machine competition (iGEM)

Established in 2004, the iGEM competition has grown to be the most successful forum for synthetic biology (SynBio) students to tackle ongoing challenges across a wide range of areas. The more than 150 startups stemming from iGEM initiatives bear testament to the impact of the competition to date (Kelwick et al., 2015; Diep et al., 2021; iGEM Foundation, 2023).

Teams are generally created around the yearly cycle of the competition and dedicate one summer (approximately 3 months of lab time) to their student-led project. Nonetheless, competition rules are sufficient flexible to allow significant institution-to-institution variation on how teams are recruited, how projects are chosen and how long students can focus on their wet lab work. Different institutions also acknowledge the student’s participation differently, from seeing iGEM as purely extracurricular voluntary participation to being an integral component of the master’s level education (Claessen and Foschepoth, 2023).

Pedagogically, iGEM is a powerful educational tool: It gives the students the opportunity to engage with a real-life problem and relevant societal stakeholder while still within the security of the university environment. The real-life scenarios lead to high student motivation and engagement with multiple stakeholders (a.k.a. human practices) offers the student opportunities for feedback on their ideas and for reflective learning. Together, this establishes ideal conditions for effective learning of molecular biology and project-specific areas (Biggs et al., 2022).

In our view, the iGEM competition has played an important role in nurturing the creativity and talent of future synthetic biologists, while framed by the advances in the field and the regular interaction with relevant stakeholders. Nonetheless, the high financial cost of student participation in the competition is unsustainable. Locally, iGEM can often be integrated into research-based education at masters’ level; with the balance between individual and group assessment one of the hardest aspects of that integration.

Alternative Synthetic Biology competitions are also emerging and rapidly maturing, e.g., The Australasian SynBio Challenge (https://www.aussynbiochallenge.org/), the Global Open Genetic Engineering Competition (https://www.gogecconference.org/) and the EUSynBioS SynBio Brewery (https://www.eusynbios.org/synbiobrewery).

## 3 Live biotherapeutic products

LBPs represent an emerging category of engineered living entities with potential clinical applications (Heavey et al., 2022). The use of SynBio tools for genetic engineering greatly enhances the ability of researchers to reprogram microbes through the availability of well-characterized genetic components and circuits (Omer et al., 2022), and novel genome modification tools, like Clustered Regularly Interspaced Short Palindromic Repeats (CRISPR) and recombineering technologies (Yoo et al., 2011). Engineering microorganisms includes enabling their selective growth within designated niches (Claesen and Fischbach, 2015; Ahan et al., 2019) and allowing them to detect and respond to physiological conditions in their surrounding environment (Hamidi Nia and Claesen, 2022). Such reprogrammed bacteria offer the potential for targeted delivery of anti-cancer therapies. Traditional chemotherapeutic agents, in contrast, often exhibit untargeted toxicity by disrupting the cell cycle and proliferation of both cancer cells and healthy cells (Omer et al., 2022).

The potential of synthetic bacterial therapies resides in their capacity to identify specific small molecules or cellular markers associated with various human diseases (Riglar and Silver, 2018). While certain detection systems can be readily adopted, either due to their natural existence or because they were designed previously, the development of many biomarker-specific sensors requires advanced techniques, such as protein design, directed evolution, site-directed mutagenesis, or domain swapping (Claesen and Fischbach, 2015). Furthermore, given the complexity of human diseases and the corresponding bacterial therapeutic devices, it is often necessary to integrate multiple input signals, evaluating various environmental cues during the diagnostic phase (Dang et al., 2023). This approach serves to minimize adverse effects on healthy cells and the resident microflora (Hamidi Nia and Claesen, 2022). Markers that indicate disease state, severity and location must be considered to effectively modulate the response of the engineered bacteria, which may include migration, the initiation of invasion, and the production of therapeutic agents. Genetic circuits in these bacteria frequently include networks of logical “AND,” “OR,” and “NOT” gates, along with promoters fine-tuned for specific purposes (Claesen and Fischbach, 2015). Another significant aspect of LBPs involves the heterologous expression of gene(s) (clusters) encoding therapeutic proteins or small molecules, or even the modulation of eukaryotic gene expression through the delivery of small RNAs (Katz et al., 2018; Holay et al., 2021).

In parallel with the therapeutic response, one of the primary concerns when utilizing genetically modified organisms is their containment (Mandell et al., 2015). To prevent horizontal gene transfer, bacterial strains used in clinical trials must lack plasmids. Plasmid-based systems can exhibit unpredictable dynamics *in vivo* over time and result in undesirable dosage effects if not adequately controlled. Therefore, it is advisable to integrate synthetic systems directly into the bacterial genome and to remove any genes involved in gene transfer. Additionally, to prevent synthetic strains from colonizing undesirable niches within the human body, it is possible to use a bacterial chassis strain that is unable to colonize the host. However, this method requires regular administration of the therapeutic bacteria, which is associated with challenges in dosage control and treatment continuity when compared to strains that are capable of colonization, environmental monitoring, and adaptive behavioral adjustment. Another strategy for containment is to incorporate a specific containment module in the synthetic therapeutic system that can deactivate or eliminate the bacterial chassis once the treatment is complete or when it moves away from its intended location in the body (Claesen and Fischbach, 2015; Mandell et al., 2015; Holay et al., 2021; Hamidi Nia and Claesen, 2022; Omer et al., 2022; Dang et al., 2023). A primary risk factor in this context is genetical instability of the LBPs due to the metabolic burden of the engineered circuits and the evolutionary pressure that is associated with this (Hamidi Nia and Claesen, 2022).

Delivering bacteria capable of sensing their local environment and responding with the controlled production of a therapeutic offers several notable advantages. Firstly, it enables localized delivery of a therapeutic, leading to lower systemic concentrations and reduced side effects and toxicity - comparable to small molecule drugs in antibody-drug conjugates. Engineered commensal strains can colonize remote niches in the human body, while sometimes in conditions that do not elicit an immune response, delivering therapeutics locally in regions that would normally be difficult to reach. In addition, they can be engineered to produce multiple synergistic compounds at the same time (Sorbara and Pamer, 2022). Despite the immense potential of these microbiota-based cell therapy systems, ensuring their safety remains a complex challenge. Secondly, the approach is thought to be cost effective: it swaps the expensive, industrial-scale production and formulation processes, for lower cost fermentation for the production of LBPs (Claesen and Fischbach, 2015; Holay et al., 2021; Dang et al., 2023). The manufacture of LBPs requires meticulous monitoring of various quality aspects, including batch uniformity, the impact of upscaling processes, and product stability. Adherence to Good Manufacturing Practices (GMPs) is imperative to guarantee the quality of LBPs throughout their pharmaceutical development and production. Proving clinical efficacy relies on stringent trials that adhere to established standards, featuring well-defined patient cohorts, treatment conditions, dosages, and validated primary endpoints. Challenges in LBP evaluation may stem from the translation of findings from animals to humans and the suitability of preclinical animal models. Safety assessments for LBPs, similar to conventional drugs, involve comprehensive risk analysis, including the identification, evaluation, and management of potential risks, subject to continuous monitoring. The unique biological properties and mode of action of live microorganisms in LBPs require specialized safety evaluation approaches compared to traditional medicines (Franciosa et al., 2023).

Multiple iGEM teams have focused on live biotherapeutic designs (Table 1). The UNI Lausanne team in 2020 engineered a therapy for colorectal cancer (CRC) using an engineered probiotic named B.O.T. This probiotic, based on *E. coli* Nissle 1917, was equipped with two plasmids: a repressilator and a sponge plasmid to enable oscillatory production of the therapeutic protein, azurin. Azurin, a copper-binding protein with a distinct blue color, was also utilized by two teams in the ‘therapeutic delivery’ category, namely, ETH Zurich in 2017 and iGEM SJTU-BioX_Shangai, 2018. Their containment strategy involved the use of two toxin/antitoxin pairs, namely, *ccdB*/*ccdA* and E2/IM2 (iGEM UNI Lausanne, 2020). A similar toxin/antitoxin approach was adopted by our iGEM KU Leuven team in 2022. We divided *ccdA*/*ccdB* across two separate plasmids, with expression of the antitoxin inhibited by an RNA thermoswitch when the cell exits the body. Our *Lactococcus*-based LBP project aimed to respond to biomarkers in a dose-dependent manner. We achieved this by utilizing both a wildtype (WT) and mutant version of the NarX-NarL two-component nitrate sensing system. The WT NarX protein could dimerize and phosphorylate the downstream effector NarL, while the mutant variant could not. Expression of the WT gene was induced by the biomarker, while the mutant was constitutively expressed. The concentration of the biomarker determined the level of downstream effector activation, subsequently influencing therapeutic protein production. Although our team did not specify biomarkers and therapeutic proteins that could have been used, our focus was on demonstrating the proof of concept (iGEM KU Leuven, 2022).

**TABLE 1:** iGEM projects focusing on detection of tumours.

Year	Team name	Project title	Project description	Reference
2010	BIOTEC Dresden	sensorBricks	Detection of CD33 and other leukemic markers to increase diagnostic stringency	iGEM BIOTEC Dresden (2010)
2012	Fatih-Medical	Cancel the cancer	Epithelial cell adhesion molecule as a pan-epithelial differentiation antigen overexpressed on the basolateral surface of most carcinomas and circulating tumor cells	iGEM Fatih-Medical (2012)
2012	BYUProvo	E. colin: A Two-Circuit System for Early Colon cancer Detection	An RNA thermosensor driven by reactive oxygen species and lactate levels for colon cancer detection	iGEM BYU Provo (2012)
2013	NU Kazakhstan	Detection of Carcinoembryonic antigen with sandwich-biosensor	Detection of carcinoembryonic antigen using ssDNA aptamers	iGEM NU Kazakhstan (2013)
2014	UFMG Brazil	The Colonyeast	Detection of long DNA for the early diagnosis of colorectal cancer	iGEM UFMG Brazil (2014)
2014	SMTexas	VOColi: Detecting Lung cancer Biomarkers	Lung cancer detection through volatile organic compounds ethanol, formaldehyde and xylene	iGEM SMTexas (2014)
2015	Harvard BioDesign	BACTOGRIP	Modification of the *E. coli* type 1 pili for specific detection and strong association to colon cancer cells	iGEM Harvard BioDesign (2015)
2015	ETH Zurich	MicroBeacon: A Microbial Beacon for cancer Detection	Detection of circulating tumor cells as a sign of metastasis using annexin V and lactate	iGEM ETH Zurich (2015)
2015	Stockholm	ABBBA The Affibody-Based Bacterial Biomarker Assay	Using HER2 as a breast cancer biomarker to develop novel chimeric receptors	iGEM Stockholm (2015)
2015	CGU Taiwan	Yes! eYE DO: Engineered Yeast and *E. coli* for Detecting Oral cancer	The use of IL-8 detection for diagnosis of oral cancer	iGEM CGU Taiwan (2015)
2016	BIT	Alarm of Breast cancer Based on Detection of MicroRNA-21 and MicroRNA-155	Breast cancer detection through micrRNA-21 and microRNA-155	iGEM BIT (2016)
2016	Stony Brook	Engineering Yeast to Develop a Novel Detection Method for the Pancreatic cancer Biomarker Glypican-1	Pancreatic cancer detection through heparan sulfate proteoglycan glypican-1 (GPC1)	iGEM Stony Brook (2016)
2017	ASIJ Tokyo	Promoting CRC Detection	Detection colorectal cancer through mutated oncogenes COX-2 and c-Myc	iGEM ASIJ Tokyo (2017)
2017	CLSB-UK	Project B.A.T.M.A.N. - Biosynthetic Applications of Toehold switches - miRNAs and non-small cell lung cancer	Detection of non-small-cell lung cancer using microRNA biomarkers hsa-mir-15b-5p and hsa-mir-27b-3p	iGEM CLSB-UK (2017)
2017	Chalmers-Gothenburg	BREATHtaking	Lung cancer detection through volatile organic compounds	iGEM Chalmers-Gothenburg (2017)
2019	Sriwijaya	CEAgar: A Reliable, Practical, and Affordable Lung cancer Diagnostic Tool	Lung cancer diagnosis using carcinoembryonic antigen (CEA)	iGEM Sriwijaya (2019)
2019	DUT China A	Cell in CELL: Encapsulation of Living CTCs using DNA Hydrogel CELL	Detection of living circulating tumor cells making use of a DNA hydrogel	iGEM DUT China A (2019)
2019	Shanghai-United	The characterization protein and early diagnosis of cervical cancer	Early detection of cervical cancer through NFX-1 detection	iGEM Shanghai-United (2019)
2019	XHD-WS-Wuhan-A	miRNA-based Detector For Gastric cancer Early Diagnosis and Future Therapy	Early detection of gastric cancer through microRNAs miR-17, miR-21, miR-196a and miR-148a expressed in patient serum	iGEM XHD-WS-Wuhan-A (2019)
2020	ZJU-China	MagHER2some	Early diagnosis of breast cancer using magnetosomes modified with anti-HER2 antibodies	iGEM ZJU China (2020)
2022	Evry_Paris-Saclay	Electricia coli	Early cancer detection relying on a toe-hold switch to detect biomarker PANTR1	iGEM Evry_Paris-Saclay (2022)
2022	Portland	Detection of Exogenous c-Myc mRNA Using Genetically Modified *E. coli*	Early cancer detection through conditionally active gRNA and CRISPR/Cas12 aimed at detecting specific mRNA sequences	iGEM Portland (2022)
2022	Wageningen_UR	Colourectal; a living diagnostic tool for colorectal cancer	Detection of lactate and matrix metalloproteinase 9 for early diagnosis of colorectal cancer	iGEM Wageningen_UR (2022)

iGEM Cornell in 2020 developed Lumicure, a system designed to identify the physical location of breast cancer metastases and enhance the therapeutic potency of primary cancer therapies when used in conjunction. They employed high lactate levels as a biomarker and implemented Trichosanthin, a ribosome-inactivating protein targeting eukaryotic cells as the therapeutic agent. Their biocontainment strategy relied on holin/antiholin proteins whose production was induced by low lactate levels (iGEM Cornell, 2020). Lastly, the LZU-China team in 2022 also utilized hypoxia, lactate, and low pH as biomarkers. They enhanced specificity and penetration with a cell adhesion module based on HlpA from *Streptococcus gallolyticus*, which is known to bind to heparan sulfate glycoprotein (HSPG) on tumor surfaces. For therapeutic purposes, their system secreted haemolysin E, CCL21, and CDD-iRGD. Haemolysin E, encoded by *hlyE* from *E. coli*, functioned as a pore-forming antitumor toxin. CCL21 recruited T-cells and dendritic cells, while CDD-iRGD triggered tumor cell apoptosis, activating the host’s immune response. To induce bacterial lysis and subsequent release of the stored therapeutic factors, they integrated the bacteriophage lysis gene (*phiX174 E*) into the circuit (iGEM LZU-CHINA, 2022).

## 4 Detection of cancer biomarkers

An important feature of a biotherapeutic cell is its ability to detect disease-specific biomarkers (Omer et al., 2022) with high specificity, selectivity, and dose-dependency (Claesen and Fischbach, 2015). Cancer biomarkers are measurable molecular cues that track the risk of cancer, its occurrence, or prognosis. These markers involve genetic variations in germline or somatic cells, epigenetic patterns, changes in transcriptional profiles, and unique proteomic signatures. The information provided by these indicators is derived from samples collected through tumour biopsies or, alternatively, from various non-invasive sources, such as blood, saliva, buccal swabs, stool, urine, and others (Sarhadi and Armengol, 2022). Over the past two decades, numerous iGEM projects have focused on this objective (Table 2). A common set of biomarkers targeted by these projects include lactate, reactive oxygen species (ROS), and heat. For instance, in 2012, BYU Provo envisioned *E. colin,* an *E. coli*-based biosensor for colon cancer detection. This biosensor employed two separate genetic circuits, one using LacZ and the other using red fluorescent protein (RFP) as reporter. The first circuit was designed to be temperature-dependent and contained a ROS-inducible promoter (*soxR*/*soxS*) driving the expression of *lacZ*. ROS is a biomarker in the TME that can originate from peroxisome activity, increased receptor signaling, oncogene activity, mitochondrial dysfunction or increased metabolic activity (Liou and Storz, 2010). However, the secondary RNA structure upstream of the *lacZ* gene only unfolded and exposed the ribosomal binding site (RBS) at a temperature of 38°C. This RNA-thermosensor was selected from a library constructed by this team using error-prone PCR. The second circuit was a lactate sensor based on the computational design of Looger et al. (2003) (iGEM BYU Provo, 2012). However, no proof of concept was reached during the time of the project.

**TABLE 2:** iGEM projects focusing on specific drug delivery to tumours.

Year	Team name	Project title	Project description	Reference
2012	HKUST-Hong Kong	B. hercules---The Terminator of Colon cancer	They express and export the anti-tumor cytokine bone morphogenetic protein 2 (BMP-2) and target it to colon tumor cells through the colon tumor homing peptide RPMrel	iGEM HKUST Hong Kong (2012)
2012	Penn	pDAWN Of A New Era: Engineering Bacterial Therapeutics	Development of an *E. coli* strain that targets HER2 overexpressing cells and secreted cytolysin A as a therapeutic protein	iGEM Penn (2012)
2013	TecMonterrey	Modular, synthetic biology approach for the development of a bacterial cancer therapy in *Escherichia coli*	A bacterial cancer therapy using *E. coli* as chassis: Toxicity module, Secretion module, Localized induction module, and Internalization module. The key components are therapeutic proteins apoptin and TRAIL and TME specific promoters HIP and nirB	iGEM Tec-Monterry (2013)
2015	Evry	The YEasT Immunotherapy project (YETI)	An engineered *S. cerevisiae* aimed at the *in vivo* treatment of melanoma was developed. The cell was equipped with immune modulators IFN γ and GM-CSF and injected into the tumor. Further, CD4^+^ and CD8^+^ T cells were elicited with a yeast antigen display system	iGEM Evry (2015)
2016	METU HS Ankara	Formation of microenvironment and production of butyrate to supress growth of cancer cells in colon	This team developed an engineered *E. coli* BL21 strain that attached to the CaCo-2 cancer cell line through a modified type I P1 structure followed by the production of butyrate, aiming to induce apoptosis in the cancer cells	iGEM METU HS Ankara (2016)
2016	Jilin China	Development of a novel cancer therapy with genetic engineered Bifidobacterium	This team constructed a recombinant plasmid containing a *Bifidobacterium* specific promoter controlling the TAT-apoptin gene expression. The transformed *Bifidobacterium* targeted tumor regions, secreting apoptin specifically toxic to tumor cells. Through TAT-mediated membrane crossing, apoptin entered solid tumor cells, inducing apoptosis	iGEM Jilin China (2016)
2016	McMasterU	Genetically engineering lactic acid bacteria for treatment of gastrointestinal tract cancers	A *Lactobacillus* strain was engineered to target gastrointestinal cancers by binding to HER2-positive tumors. Upon binding, IL-2 production is induced through a QS-based mechanism	iGEM McMasterU (2016)
2016	Duesseldorf	Optogenetic Induction of Apoptosis in cancer Cells	This team developed an optogenetic circuit utilizing phytochrome B and LOV2	iGEM Duesseldorf (2016)
2017	TP-CC San Diego	cancer Research Utilizing CRISPR based ecDNA Modification	Therapy based in CRISPR/Cas system that targets extrachromosomal DNA needed for oncogene distribution	iGEM TP-CC San Diego (2017)
2017	ColumbiaNYC	SilenshR: Bacteria-Mediated Oncogene Silencing as Living cancer Therapeutic	Recombinant *E. coli* cells were equipped with an RNAi gene therapy. After mammalian cell invasion, they deliver the shRNA payload that enables the expression of tyrosine kinase receptor EGFR and transcription factor c-Myc	iGEM Columbia NYC (2017)
2017	Freiburg	CARtel - Chimeric Antigen Receptor on T cells Expressed Locally in the tumor Microenvironment	This team modified the CAR-T cell therapy to only be activated in the TME. To this end, they used an AND gate responding to hypoxia, low pH and Vascular Endothelial Growth Factor	iGEM Freiburg (2017)
2017	ETH Zurich	CATE - cancer-Targeting *E. coli*	This team implemented an AND gate responding to lactate levels and live biotherapeutic population threshold. Upon activation, the therapeutic protein azurin is expressed	iGEM ETH Zurich (2017)
2018	LZU-CHINA	New therapy for gastric cancer based on TIL cells-exosomes mechanism	An exosome-based gastric cancer therapy that delivers microRNA.	iGEM LZU-China (2018)
2018	UPF CRG Barcelona	Probiotics to fight metastasis: Engineering *E. coli* to regulate fatty acid metabolism	An engineered probiotic aimed at reducing long chain fatty acid concentration in the gut to reduce metastasis	iGEM UPF CRG Barcelona (2018)
2018	HZAU-China	Pyroptosis: a new approach for cancer therapy	*Salmonella* was redesigned to act as a delivery vehicle targeting tumor cells and replicate in their cytoplasm. The bacterial expression of the N-terminal domain of gasdermin D induced bacterial lysis and release of gasdermin D into the cytoplasm of tumor cell followed by pyroptosis to the tumor cell	iGEM HZAU-China (2018)
2019	SMMU-China	Wukong: an Engineered Theranostics based on Synthetic Immune Cells	A novel Engineered Theranostics with the core device in which the CAR-immune cells were reprogramed to co-evolve with tumor-antigens and to send secondary signals to trigger custom-designed external devices	iGEM SMMU-China (2019)
2019	NEFU China	Bacterium Oncologists: Guide Us to cancer!	This *E. coli* Nissle 1917 live biotherapeutic responds to alterations in uric acid levels by expressing an anticancer drug	iGEM NEFU China (2019)
2019	IISER Tirupati	A Potential Probiotic for Targeted Immunotherapy against Colon cancer	*E. coli* was engineered to treat colon cancer. It expresses a homing peptide on the cells fimbriae. After association, IL-12 is expressed under the control of a lactate-sensitive promoter	iGEM IISER Tirupati (2019)
2022	IISER_TVM	Duonco: A dual nanovesicle drug delivery system targeting breast cancer	Duonco is a live biotherapeutic product targeting HER2 and CX3CR1, two breast cancer surface biomarkers. After association, the cell is engineered to produce outer membrane vesicles containing anticancer drugs	iGEM IISER_TVM (2022)

The MicroBeacon project from ETH Zurich in 2015 was also designed to sense lactate and temperature, but through different mechanisms. It aimed to detect circulating tumor cells (CTCs) through elevated lactate production and sensitivity to soluble TNF-related apoptosis-inducing ligand (sTRAIL). Treatment of patients’ blood samples with sTRAIL led to cancer cells producing phosphatidylserine, which was consequently targeted by annexin V on the MicroBeacons. Furthermore, the elevated lactate levels triggered a quorum sensing (QS) circuit in *E. coli* cells, which, together with the annexin V interaction, induced green fluorescent protein (GFP) expression. This process was performed in microfluidic chips, using water-in-oil emulsion droplets (iGEM ETH Zurich, 2015).

Other projects that have aimed to detect CTC include “Cancel the cancer,” which targeted the epithelial cell adhesion molecule (EpCAM) biomarker (iGEM Fatih-Medical, 2012), and “cell in CELL” by DUT China A in 2019. The latter project designed a multifunctional DNA hydrogel in a three-step process. First, fluorescently labelled single-stranded DNA (ssDNA) aptamers were used for precise targeting. These aptamers then underwent a conformational change, revealing sticky ends that facilitated the binding of complementary sticky-end ssDNA molecules. This interaction initiated a series of molecular amplification processes, starting with rolling circle amplification and followed by multi-primed chain amplification, which resulted in the formation of a hydrogel structure around the captured CTCs. This concept was proven *in vitro* using HeLa and MCF-7 cells (iGEM DUT China A, 2019).

Wageningen_UR applied temperature differences as a biocontainment strategy in their Colourectal project, combining it with multiple CRC biomarkers. Lactate was detected with the lactate-inducible ALPaGA promoter, well-suited for the anoxic and glucose-rich environment of the colon. Detection of carcinoembryonic antigen-related cell adhesion molecule 6 (CEACAM6) and integrin α5β1 was used for tumor colonization. Modified chromoproteins served as reporter compounds and could only be activated in the presence of matrix metalloproteinase 9, which served as another CRC biomarker. They were able to prove the success of their detection module. However, it was not able to distinguish between healthy and cancerous lactate levels. Furthermore, they were not able to completely optimize the biosafety and reporter modules during the timespan of their project (iGEM Wageningen_UR, 2022). Carcinoembryonic antigen (CEA) was also employed as a cancer biomarker in other projects, such as iGEM NU Kazakhstan (2013) and iGEM Sriwijaya (2019).

Magnetotactic bacteria have a natural ability to form magnetosomes, which enable them to align with a magnetic field and navigate in response to oxygen gradients. This unique feature offers the significant advantage of controlling the bacteria remotely and non-invasively via magnetic fields (Yan et al., 2017). The ZJU China 2020 team assembled single-chain variable fragments (scFv)-magnetosomes, called MagHER2somes, as a novel contrast agent. This agent specifically targeted HER2-positive breast cancer cells by displaying anti-HER2 scFv via the anchor protein MamC on their Shuffle strain (iGEM ZJU China, 2020). HER2 has also been used by iGEM Stockholm (2015) as a breast cancer biomarker.

The iGEM community has also explored non-protein biomarkers, such as RNA. iGEM CLSB-UK (2017) used microRNA (miRNA) 15b-5p and miRNA 27b-3p for non-small cell lung cancer (NSCLC) detection. They developed a cell-free biosensor for NSCLC in which toehold switches were designed to activate the expression of reporter genes upon binding of the miRNA. This NSCLC-specific miRNA interacts and unfolds the toehold switch, which exposes the RBS and allows expression of the reporter gene (iGEM CLSB-UK, 2017). iGEM XHD-WS-Wuhan-A (2019) employed miRNA 21, 17, 196a, and 148a as gastric cancer biomarkers. Further, the Portland 2022 team focused on a segment of c-Myc mRNA, suitable for a non-invasive multi-cancer early detection (MCED) test as it could be identified in plasma (iGEM Portland, 2022).

The 2014 UFMG Brazil team developed Colonyeast, a *Saccharomyces cerevisiae*-based sensor designed to detect long DNA molecules in stool samples from CRC patients, using a split mCherry system brought together by DNA-binding domains (iGEM UFMG Brazil, 2014). Harvard BioDesign in 2015 enhanced *E. coli*’s type I pili by eliminating nonspecific mannose binding and replacing it with a HT29 colon carcinoma-specific homing peptide RPMrel from the TumorHoPe database (Kelly and Jones, 2003), previously used by iGEM team HKUST Hong Kong 2012 (iGEM Harvard BioDesign, 2015). Finally, additional biomarkers, such as CD33 for acute myeloid leukemia (iGEM BIOTEC Dresden, 2010), volatile organic compounds (xylene, ethanol, formaldehyde, cyclohexane) for lung cancer diagnosis (iGEM SMTexas, 2014; iGEM Bilkent-UNAMBG, 2016; iGEM Chalmers-Gothenburg, 2017), IL-8 (iGEM CGU Taiwan, 2015), glypican 1 (iGEM Stony Brook, 2016), β-catechin (iGEM ASIJ Tokyo, 2017), and PANTR1 (iGEM Evry_Paris-Saclay, 2022) have been employed for detecting various other cancers. The variability of biomarkers is wide and they can be categorized as protein, nucleic acid, small molecule and primary metabolite biomarkers. Nonetheless, these are suspected to only be a small fraction of the known and characterized biomarkers.

## 5 Delivery of a biotherapeutic compound

Some iGEM projects focused on delivery of a therapeutic compound by the LBP (Table 3). RPMrel, the homing peptide mentioned earlier, was first explored by the HKUST-Hong Kong 2012 team to enable targeted drug delivery. They developed *B. hercules*, an engineered *Bacillus subtilis* strain to treat colon cancer. The application involved oral administration of this engineered bacterium, which was designed to survive passage through the digestive tract. The LBP targeted cancer cells via RPMrel, and the timing of drug release was controlled by a xylose-inducible promoter. Xylose was aimed to be provided to the gut in oral capsules when the LPB had successfully localized to the colon tumor. The therapeutic protein delivered was bone morphogenetic protein 2 (BMP 2), which induces cell cycle arrest in the G1 phase and apoptosis in tumorous colon epithelial cells. Other tested therapies aimed at inducing cell cycle arrest and/or apoptosis include butyrate delivery (iGEM METU HS Ankara, 2016) and the modification of *bax* and *bcl-2* expression in tumor cells using a CRISPR interference (CRISPRi) and CRISPR activation (CRISPRa) system (iGEM SCUT-China B, 2016). CRISPR technology was also used by the high school team TP-CC San Diego to target double-stranded extrachromosomal DNA (ecDNA) carrying oncogenes. The CRISPR/CRISPR-associated nuclease 9 (CRISPR/Cas9) system is a powerful genome editing tool that introduces double-stranded breaks at targeted locations (Wang et al., 2022). In contrast, CRISPRi employs a catalytically inactive Cas9 (dCas9), which, in complex with a single guide RNA (sgRNA), creates steric hindrance, thereby repressing the gene of interest (Larson et al., 2013). Conversely, CRISPRa uses dCas9 in conjunction with a transcriptional activator, resulting in the upregulation of the gene of interest (Heidersbach et al., 2023).

**TABLE 3:** iGEM projects focusing on biocontainment strategies for live biotherapeutic cells.

Year	Team name	Project title	Project description	Reference
2014	Cooper Union	Micro-Toolbox: Open Source Solutions for DNA Synthesis, Biosafety and SynBio Education	A yeast-based chassis with a programmable lifespan based on telomeres	iGEM Cooper Union (2014)
2017	NUS Singapore	Making engineering of customised kill switches easier!	Development of a library of sensors, combined with killing and verification modules	iGEM NUS Singapore (2017)
2017	Hong Kong HKUST	Genetic Containment Strategy: Preventing the Replication of unintentionally released Genetically Modified Materials through Recombinase-based Deletion	A safety circuit that prevents the genetically modified segment to replicate in the host organism	iGEM HKUST Hong Kong (2017)
2018	SYSU-CHINA	Braking Bad--Torwards a safer CAR-T therapy	Enhancement of the CAR-T therapy through a reversible safe switch that allows up- and downregulation of CAR molecules through endosomal recycling inhibition	iGEM SYSU-China (2018)
2020	Cornell	Lumicure	An *E. coli*-based therapy employing a lactate-induced toxin-antitoxin system GhoS/GhoT as containment strategy	iGEM Cornell (2020)

In 2013, Tec-Monterrey, the Latin America Grand Prize winner, earned four additional awards for developing four modules for a drug delivery system designed to induce apoptosis in cancer cells. iGEM Tec-Monterry (2014) successfully integrated these modules into a comprehensive framework. The four modules covered various functions: the cytotoxicity module enabled the production of the therapeutic proteins TRIAL and apoptin. The latter was modified with the TAT-peptide to enable entry into tumor cells (internalization module). The secretion module allowed the therapeutic proteins to be secreted through a hemolysin secretory mechanism. Finally, the localized induction module ensured the specific production of these therapeutic proteins in proximity to tumor cells by regulating their expression through the hypoxic promoters HIP and nirB. Another team building on the modular design of Tec-Monterrey 2013 was Jilin China 2016, who specifically focused on the TAT-apoptin fusion as a therapeutic protein. They utilized a *Bifidobacterium* strain as a chassis and tested different signal peptides to optimize the secretion of the drug.

In 2019, iGEM Hong Kong HKU (2019) adopted an engineered *Salmonella enterica* serovar *typhimurium* strain as a chassis for biotherapeutic delivery. They utilized an *E. coli* system, previously developed by the Etherno team (iGEM HKU Hong Kong, 2018), that is capable of producing two specific DNA nanostructures for targeting an epithelial cell adhesion molecule in liver cancer stem cells and nucleolin on the surface of cancer cells, respectively. The engineered *S. enterica* serovar typhimurium SL7207 possessed three key properties: The system delivered doxorubicin (a topoisomerase II inhibitor commonly used as a chemotherapeutic agent against a range of different cancers), as well as an artificial miRNA as a DNA tetrahedral drug carrier. Several teams have focused on RNA-based therapies, such as iGEM NJU-China (2016), who developed a small interfering RNA (siRNA) exosome specifically targeting integrin to modify mutated KRAS expression; KRAS being one of the most commonly mutated oncogenes in lung cancer and with integrin used as biomarker. Columbia NYC 2017 also developed an RNAi-based therapy targeting c-Myc and epidermal growth factor receptor (EGFR) with short hairpin RNA (shRNA). The process of bacterial uptake by mammalian cells and the subsequent breakdown of endosomes was facilitated by a quorum sensing-inducible Invasin-HlyA operon.

To enhance therapy specificity, IISER TVM 2022 introduced Duonco, a unique drug delivery system designed to combat breast cancer. Duonco primarily targeted HER2 and CX3CR1, both frequently overexpressed in breast cancer. The team modified *E. coli* bacteria to produce two distinct types of outer membrane vesicles (OMVs). These OMVs were specifically designed to interact with HER2^+^ cancer cells through engineered affibodies (ZHER2:342). Moreover, the OMVs can transport chemotherapeutic prodrugs and the enzymes required for their activation. Employing OMVs for targeted drug delivery offers several advantages, such as the ability to easily customize them with foreign epitopes and transport diverse payloads. Additionally, OMVs protect their cargo from external factors and dilution, and they possess intrinsic immunostimulatory properties, thereby enhancing the ability of the innate immune system to target cancer cells (Grandi et al., 2017). The prodrug-enzyme system consisted of cytosine deaminase, which converted 5-fluorocytosine (5FC) to 5-fluorouracil (5FU). The activated 5FU could traverse into neighboring cells to exert its anticancer effects through the inhibition of thymidylate synthase and its incorporation into RNA and DNA. Even a small amount of the activated drug, when injected at the core of the tumor, was expected to significantly reduce tumor size (Longley et al., 2003).

Numerous iGEM projects were centered around immune-based therapies. In 2019, the SMMU-China team developed “Wukong,” synthetic natural killer (NK) cells encoding designed synNotch and chimeric antigen receptors (CARs). Team Evry 2015 aimed to recruit CD4^+^ and CD8^+^ T cells, while McMasterU 2016 targeted HER2 for the specific delivery of the T-cell activating cytokine IL-2. A similar approach was employed by IISER Tirupati 2019, who used an engineered *E. coli* strain to produce IL-12 induced by factors in the TME. Finally, the Freiburg 2017 team designed a CAR T cell line that expressed CARs only when detecting compounds specific to the TME.

## 6 Biocontainment strategies

The safe use of engineered clinical probiotics involves important biocontainment and biosafety considerations. These aspects are essential for obtaining approval for clinical testing and application of therapeutic products (Riglar and Silver, 2018). Biocontainment measures are designed to prevent unintended escape of engineered bacteria into the environment. They encompass various strategies, such as preventing bacterial transfer between individuals, controlling bacterial growth and the expression of therapeutic agents, and limiting gene transfer to and from the engineered bacterial strain (Charbonneau et al., 2020). Approaches include the use of auxotrophies or the use of non-colonizing strains to restrict their residence time. It is important to note that biocontainment systems can impose a burden on the metabolism of the engineered host cells or cause background toxicity, which can hinder the performance of the cells and compromise the biocontainment mechanism. These systems therefore require tight regulation under conditions that permit their activity (Wook Lee et al., 2018).

So far, only four iGEM projects have concentrated on the development of biocontainment strategies (Table 4). The team of Cooper Union 2014 devised a strain of *S. cerevisiae*, named the Super Safety Strain, with a controlled lifespan. To achieve this, they disrupted genes responsible for ever-shortening telomeres 2 (EST2) and RAD52, a protein involved in homologous recombination. EST2 is a crucial subunit of yeast telomerase, and its deletion renders the enzyme non-functional. However, yeast cells possess an alternative mechanism, called Alternative Lengthening of Telomeres (ALT), which elongates telomeres through homologous recombination. To deactivate this system, they suppressed the expression of RAD52 (iGEM Cooper Union, 2014). Another approach involved the separation of the toxin/antitoxin system E2/IM2 across two distinct plasmids in *E. coli*. The expression of the antitoxin is inhibited by specific environmental cues, such as the low temperature and low phosphate levels encountered outside the host, as demonstrated by the NUS Singapore 2017 team (iGEM NUS Singapore, 2017).

**TABLE 4:** iGEM projects including detection, drug delivery and biocontainment in their project design.

Year	Team name	Project title	Project description	Reference
2017	Newcastle	Sensynova - a new era of biosensors	Propose a modular, multicellular development platform where new sensors are developed by mixing proportions of cells that are able to communicate through small molecules	iGEM Newcastle (2017)
2017	McMaster II	C12 Mediated cancer Treatment: Dual Functionality as a Cytotoxic and Signalling Agent	Live biotherapeutic product that expressed acyl-homoserine lactone C12 upon detection of hypoxia and reduced pH. C12 is known for its induction of cell apoptosis through a Bcl-2 independent pathway. Further, the quorum sensing capacities of C12 have been employed as a containment module	iGEM McMaster II (2017)
2020	UNILausanne	B.O.T: Bacterial Oscillation Therapy	*E. coli* Nissle 1917 was engineered as a live biotherapeutic product to target colorectal cancer based on chronotherapy	iGEM UNI Lausanne (2020)
2022	KU_Leuven	Dose-dependent colorectal cancer biosensor and therapy delivery system	We developed a *Lactococcus*-based live biotherapeutic employing a Nax-NarL-based sensing system and temperature-sensitive riboswitches as biocontainment strategy. These switches inactive that anti-toxin part of the type II toxin-antitoxin system upon a temperature drop	iGEM KU Leuven (2022)
2022	LZU-CHINA	Targeted Treatment of Colorectal Cancer with Gene-Editing Probiotics	Development of an engineered probiotic for the targeted treatment of colorectal cancer. They built three detection modules aimed at hypoxia, low pH and lactic acid concentrations. Further, they implemented an adhesion module using the *Streptococcus* gallolyticus HlpA gene which interacts with heparan sulfate glycoprotein on the tumor surface. Finally, hemolysin E, CCL21 and CDD-iRGD (Bit1 fusion protein of cell death domain and tumor perforin) are used as therapeutic proteins and bacteriophage lysis gene phiX174 E was employed as biocontainment strategy	iGEM LZU-CHINA (2022)

HKUST Hong Kong 2017 employed a more elaborate biocontainment strategy involving three modules. The sensing module was designed to detect the level of acyl homoserine lactones (AHLs) within the cell. Its primary role was to amplify the AHL signal using a positive feedback loop. The time control module introduced a time delay in the cellular response to the AHL signal. It regulated the translation of two repressor genes located downstream of the sensing promoter. The activation of lambda cI repressor inhibited the PhlF repressor, which consequently allowed for the activation of Cre recombinase expression in the subsequent recombination module. The signal transduction through the time control module allowed the sensing module enough time to amplify the AHL signal through its positive feedback loop. In the final recombination module, Cre recombinase spatially separated the origin of replication from the rest of the plasmid as proof of concept. As a result, plasmids that underwent recombination lost their ability to replicate. Over time, this led to a gradual loss of the target gene within the cell population (iGEM HKUST Hong Kong, 2017).

Lastly, iGEM SYSU-China (2018) focused on enhancing the safety of CAR-T therapy. Administering CAR-T cells without effective control mechanisms carries significant risks of severe adverse effects, especially during clinical trials. While traditional suicide switches have been used to manage these adverse effects, they induce a complete halt to the costly treatment. This interruption leads to a burdensome repetition of the treatment process for patients, both physically and financially. The team introduced a reversible safety switch, CAR BRAKE, controllable by small molecules. This mechanism relies on the expression of the U24 gene from human herpesvirus 6A, regulated by the tet-ON promoter. U24 operates by downregulating CAR molecules on the cell surface through the inhibition of endosomal recycling. This reduction in CARs impedes the functioning of CAR T cells. Importantly, this downregulation can be reversed by removing doxycycline, highlighting the switch’s reversibility (iGEM SYSU-China, 2018).

## 7 Challenges in translation of LBP research

The engineering of LBPs has been the focus of many iGEM projects, but none have progressed beyond proof-of-concept towards clinical validation. The timeframe of the iGEM competition and the research costs for testing LBPs in a clinically-relevant context make it impossible for iGEM teams to take any LBP beyond the proof-of-concept stage. In addition, many teams encountered significant challenges in implementing their circuits, making even proof-of-concept LBPs a challenging goal. Advancing iGEM projects beyond the competition remains a possibility if there is sufficient academic and student interest, and this has been successfully achieved in some projects. However, none of these were LBP-focused projects, and there are multiple reasons why this translation is challenging.

Even academic and industrial researchers face difficulties, as evidenced by the low number of projects in late clinical trials, extensively reviewed by Murali and Mansell (2024). Developing LBPs as medical drugs is complex, involving scientific, regulatory, manufacturing, and market acceptance challenges. Scientific challenges are associated with the complexity of biological systems. LBPs are metabolically active organisms that can help re-establish balance and reduce dysbiosis by modulating the host microbiome, leading to downstream effects. Interactions with the host’s immune system and metabolic processes are complex and not fully understood, making predictions and consistent outcomes difficult (Roslan et al., 2023). Additionally, biological systems are inherently variable, with organisms behaving differently due to genetic and microbiota variations. As such, *in vitro* and animal model data often fail to translate into safe and efficacious human treatments, necessitating novel model systems to better characterize LBPs before clinical testing (Paquet et al., 2021).

A significant limitation in this field is the lack of strategies for controlled delivery of LBPs that ensure therapeutically optimal concentrations. Strategies to overcome these challenges were reviewed by Heavey et al. (2022). Furthermore, establishing a quantifiable relationship between LBP effectors and the underlying mechanisms of disease is crucial. However, there is a notable scarcity of disease- and effector-related biomarkers necessary to construct a dose-dependent response model (Charbonneau et al., 2020). A comprehensive understanding of the mechanisms of action would be beneficial, though it may be influenced by numerous external factors or depend on the target population and therapeutic targets. Achieving long-term colonization of LBPs could be advantageous, allowing for continuous disease monitoring and delivery of therapeutic effectors, reducing the need for repeated dosing, and enabling lower efficacious bacterial dose levels. This approach could enhance patient receptivity and compliance, although biocontainment concerns must be addressed (Charbonneau et al., 2020; Cordaillat-Simmons et al., 2020).

Biocontainment remains a critical challenge in the field. Novel approaches, such as the implementation of auxotrophies to restrict the proliferation of engineered strains to specific environments, are currently under investigation. Biocontainment strategies encompass mechanisms which inhibit unintended spread by responding to particular environmental cues. These methodologies address essential safety issues and are instrumental in fostering responsible and controlled progress in LBP research (Charbonneau et al., 2020; Rutter et al., 2022; Roslan et al., 2023). However, multiple orthogonal biocontainment strategies will need to be implemented to ensure sufficient biocontrol and safety.

The regulatory landscape for LBPs is complex and many reviews have been written about this (Dreher-Lesnick et al., 2017; Cordaillat-Simmons et al., 2020; Paquet et al., 2021). Regulatory approval must consider broader impacts, including environmental and societal factors, as highlighted by Charbonneau et al. (2020). Manufacturing facilities must produce standardized, high-quality LBPs at scale. This poses significant challenges on the industrial process, including scalability, maintaining viability, purity, consistency, and preventing contamination and batch-to-batch variation (Cordaillat-Simmons et al., 2020).

Despite these obstacles, LBPs exhibit considerable promise in meeting previously unaddressed medical needs. Progress in biological technologies is expected to enhance strategies for disease prevention and treatment by introducing innovative bacterial tools and refined therapeutic approaches, thereby mitigating safety concerns. As LBPs near clinical application, it will be important to observe societal responses to these technologies. The ongoing investigation and refinement of biocontainment techniques have significant implications for the development and acceptance of LBPs in clinical settings.

## 8 Conclusion and future perspectives

The field of LBPs represents a promising avenue for the development of novel clinical interventions. LBPs, engineered with synthetic biology tools, hold significant potential for various clinical applications, especially in the context of cancer therapy (Hanahan and Weinberg, 2000; Hanahan and Weinberg, 2011; Claesen and Fischbach, 2015; Riglar and Silver, 2018; Charbonneau et al., 2020). Key themes in that area have been the focus of iGEM projects, including the detection of disease-specific biomarkers, the delivery of biotherapeutic compounds, and the implementation of biocontainment strategies.

While several iGEM projects have demonstrated the potential of LBPs in biomarker detection and drug delivery, some knowledge gaps remain. For example, a limited number of projects so far have explored the significance of customizable payload delivery and the potential for associated side effects (Holay et al., 2021). Furthermore, specific therapeutic approaches, such as the utilization of nanobodies and bactofection, have remained relatively unexplored within the scope of iGEM projects. Bactofection, a strategy involving the use of bacteria to facilitate the transfer of compounds into mammalian cells, has demonstrated promise in delivering genetic materials for anti-cancer therapy (Hosseinidoust et al., 2016). This innovative approach can also be leveraged to introduce genes with immunomodulatory properties into cancer cells (Pilgrim et al., 2003). Notably, bacteria themselves are known to trigger an immunostimulatory response, contributing to immune upregulation. However, importantly, this response is only acceptable in the TME (Baban et al., 2010). Furthermore, extensive research has been conducted on the use of bacteria in vaccine design, indicating their potential utility in this context (Kroll et al., 2019). While many iGEM projects have focused on immune modulation as a strategy for cancer therapy, primarily through adaptations of chimeric antigen receptor T cell therapy, it is important to acknowledge that a substantial portion of patients do not experience the anticipated benefits from such approaches (Gong et al., 2018).

Biocontainment strategies are crucial for ensuring the safety and controlled use of LBPs in clinical settings. While clinical probiotics based on engineered microbes offer significant therapeutic potential, they also raise concerns about environmental release and uncontrolled proliferation. To address these challenges, iGEM teams have developed innovative biocontainment measures, such as reversible safety switches, toxin/antitoxin systems, and temperature-dependent regulation. These strategies aim to prevent unintended bacterial escape and loss of control over engineered microbes within the host. Bacterial encapsulation remains a relatively unexplored domain in iGEM projects. Traditional encapsulation techniques involve the application of materials, such as alginate, polymers, or hydrogels (Hsu et al., 2020). To ensure the optimal functionality of therapeutic interventions, it is imperative to facilitate the translocation of metabolites across hydrogel pores, which can be intricately engineered to selectively impede the passage of bacteria themselves (Bahram et al., 2016). Hydrogels, renowned for their biocompatibility and versatile properties, including porosity, biodegradability, mechanical flexibility, and chemical adaptability, enjoy widespread utilization in both environmental and biomedical applications (Hamidi Nia and Claesen, 2022).

While the majority of iGEM projects have primarily focused on *in vivo* applications within the gut, it is foreseeable that future endeavors will target the oral cavity and skin as emerging platforms (Hamidi Nia and Claesen, 2022). *Lactococcus lactis*, although frequently employed in gut-related contexts, holds promise as a compelling candidate for applications within the oral cavity, alongside *Streptococcus salivarius* K12 (Burton et al., 2006; Caluwaerts et al., 2010). For applications involving the skin, commensal species such as *Corynebacterium* and *Staphylococcus epidermidis* represent well-suited contenders (Claesen and Fischbach, 2015).

Overall, the development of LBPs as a therapeutic platform represents a promising frontier in synthetic biology and clinical medicine. As the field continues to evolve, addressing challenges related to stability, safety, and regulatory approval will be essential. The continued and systematic exploration of the area is essential to disseminate innovative ideas, train future researchers (and industrialists), as well as bring a significant impact to the lives of patients.
